# Reprogramming the Evolution of High-Risk Prostate Cancer: Multidisciplinary Strategies to Delay Castration Resistance

**DOI:** 10.3390/jcm15166488

**Published:** 2026-08-21

**Authors:** Younghun Sim, Jae Won Choi, Dong Seob Kim, Jeong Hyun Kim, Sung Goo Yoon, Jung Ki Jo

**Affiliations:** 1 Department of Urology, Hanyang University Seoul Hospital, Seoul 04763, Republic of Korea; subika@hanyang.ac.kr (Y.S.); mj2118@naver.com (J.W.C.); peter1114@gmail.com (D.S.K.); 2 Department of Urology, Kangwon National University School of Medicine, Chuncheon 24289, Republic of Korea; urodr348@kangwon.ac.kr; 3 Department of Urology, College of Medicine, Hanyang University, Seoul 04763, Republic of Korea

**Keywords:** prostate cancer, castration-resistant prostate cancer, androgen deprivation therapy, multimodal therapy, tumor evolution, lineage plasticity, PSMA radioligand therapy

## Abstract

**Background/Objectives**: High-risk prostate cancer is a biologically heterogeneous group of tumors carrying a substantial risk of progression to lethal, castration-resistant disease. Although androgen deprivation therapy (ADT) remains the therapeutic backbone, nearly all advanced disease eventually progresses to castration-resistant prostate cancer (CRPC) through Darwinian clonal evolution under sustained therapeutic pressure. This narrative review reframes high-risk prostate cancer management as an effort to reprogram the evolutionary trajectory and delay castration resistance, addressing current risk stratification, androgen receptor (AR)-dependent and AR-independent mechanisms of resistance, and multidisciplinary strategies that modify selective pressure. **Methods**: A narrative review of the literature was conducted, including peer-reviewed studies, pivotal phase III trial reports, and current clinical practice guidelines indexed in PubMed, Scopus, and Web of Science up to 2026. Sources on high-risk and castration-resistant prostate cancer, the biology of treatment resistance, and multidisciplinary treatment intensification were selected and synthesized. **Results**: Treatment intensification has been extended to high-risk biochemical recurrence (EMBARK), directed by biomarkers in PTEN-deficient disease (CAPItello-281), and moved earlier through prostate-specific membrane antigen (PSMA)-targeted radioligand therapy (PSMAfore, PSMAddition). In localized disease, effective AR-pathway intensification with abiraterone (STAMPEDE) contrasts with the failure of chemotherapy (PEACE-2) and enzalutamide (ENZARAD). Emerging therapies targeting lineage plasticity exploit its dynamic and potentially reversible biology, raising the prospect of reversing established resistance rather than merely delaying it. CAPItello-281 and PSMAddition have immature overall survival data and are not yet standard of care. **Conclusions**: Coordinated multidisciplinary care, matched to the disease stage and molecular context, offers a realistic path to delay castration resistance and improve survival.

## 1. Introduction

Prostate cancer is one of the most frequently diagnosed malignancies in men worldwide and is a leading cause of cancer-related deaths. Historically less common in Asian than in Western populations, it is now rising rapidly across Asia, including South Korea, as populations age and diets westernize [1,2]. High-risk and locally advanced tumors make up a substantial share of new diagnoses, approximately 15–30%, depending on the criteria applied; however, they pose a disproportionate clinical challenge: they progress early, metastasize, and drive much of prostate-cancer mortality [3]. For men with advanced disease, androgen deprivation therapy (ADT) has been the cornerstone of treatment for more than eight decades, reflecting the central dependence of prostate cancer on androgen receptor (AR) signaling. However, that benefit is finite. Under sustained androgen suppression, the disease almost invariably adapts and progresses to castration-resistant prostate cancer (CRPC), which is refractory to treatment and associated with poor outcomes [4].

Castration resistance is not a single biochemical switch. In fact, it is a branched Darwinian process: sustained therapeutic pressure selects for pre-existing and newly acquired resistant clones, expands AR-amplified and AR-splice-variant populations, and, in a subset of tumors, promotes lineage plasticity toward AR-independent, neuroendocrine phenotypes [4]. Seen this way, the therapeutic question changes. It is no longer only how to suppress the androgen axis, but how to shape the disease’s evolution and determine which agents, and in what combination and sequence, will limit the selection of resistant clones and delay, or occasionally prevent, castration resistance.

These shifts are already visible in the clinic. Upfront intensification outperforms sequential monotherapy in metastatic hormone-sensitive disease [5,6]; combining local and systemic therapy improves survival in high-risk disease [7]; and biomarker-defined subgroups now allow targeted intervention [8]. The pace has been striking. In just two to three years, intensification has moved earlier—into high-risk biochemical recurrence—been refined by molecular selection, and been extended by prostate-specific membrane antigen (PSMA)-targeted radioligand therapy, even as negative trials in very-high-risk localized disease have clarified where and with which agents it helps. Realizing these gains requires urology, radiation oncology, medical oncology, pathology, radiology, and supportive care to act as a genuine multidisciplinary team, rather than a sequence of separate consultations.

This review treats high-risk prostate cancer as a problem of reprogramming disease evolution to delay castration resistance. The scope of this review is deliberately set along the whole disease continuum—localized high-risk disease, biochemical recurrence, metastatic hormone-sensitive disease, and non-metastatic and metastatic castration-resistant disease—because castration resistance develops across these settings rather than at any single point within them. The term high-risk prostate cancer is therefore used throughout to denote this evolutionary trajectory rather than localized high-risk disease alone. Nonetheless, the decisions that each setting demands differ enough that we keep them distinct. We define and stratify high-risk disease; set out AR-dependent and AR-independent mechanisms of resistance, with particular attention to lineage plasticity; and trace how systemic therapy, radiation, surgery, and supportive care apply across the continuum. We draw throughout on the practice-changing trials of 2024–2026, separating established evidence from investigational evidence, and close with emerging therapies and biomarker-driven approaches.

## 2. Materials and Methods

This narrative review synthesized peer-reviewed clinical trials, current clinical practice guidelines, and mechanistic studies relevant to treatment resistance and intensification in high-risk prostate cancer. PubMed, Scopus, and Web of Science were searched for English-language articles published through early 2026. Search terms combined terms for the disease (prostate cancer; high-risk; locally advanced; castration-resistant) with terms for its biology and treatment (androgen receptor; lineage plasticity; neuroendocrine differentiation; treatment intensification; androgen deprivation therapy; PSMA; radioligand therapy; PARP inhibitor). Reference lists of key articles and recent reviews were hand-searched, and 2024–2026 proceedings of major oncology and urology meetings were screened for trial data not yet published in full. Randomized phase III trials and guideline statements were prioritized, together with translational studies bearing directly on AR signaling, lineage plasticity, and biomarker-directed therapy.

## 3. High-Risk Prostate Cancer: Definition and Biological Heterogeneity

### 3.1. Defining and Stratifying High-Risk Disease

High-risk prostate cancer is most commonly defined by criteria derived from the D’Amico classification and incorporated, with modifications, into the National Comprehensive Cancer Network (NCCN) [9] and European Association of Urology (EAU) [10] guidelines. Localized disease is generally classified as high-risk when any one of three features is present: a serum prostate-specific antigen (PSA) level > 20 ng/mL, International Society of Urological Pathology (ISUP) grade group 4–5 (Gleason score 8–10), or an advanced clinical stage. The stage threshold is cT2c or higher in the D’Amico scheme and cT3a in the NCCN, which otherwise applies the same PSA and grade thresholds. The EAU defines high-risk localized disease by grade group 4–5 or PSA > 20 ng/mL and classifies clinical stage cT3–cT4 or regional nodal disease (cN1) as locally advanced disease, which falls within the broader high-risk category (Table 1). The NCCN further distinguishes a very-high-risk subgroup: clinical stage cT3b–cT4, primary Gleason pattern 5, more than four biopsy cores of grade group 4–5, or multiple high-risk features [9].

The burden of high-risk disease is not evenly distributed. In a Surveillance, Epidemiology, and End Results analysis of 383,039 men, the proportion with high-risk cancer at diagnosis rose with age, from 14.3% at ages 50–54 to 22.4% at ages 70–74, and 38.7% at ages 80–84 [11]. Black men are also affected disproportionately. In a California registry study of 23,555 Black and 146,889 non-Hispanic White men, Black men had 67% higher adjusted odds of presenting with a PSA above 20 ng/mL. Roughly one quarter of that disparity was attributable to neighborhood socioeconomic status [12].

These definitions, although clinically useful, group together patients with markedly different prognoses. They rely on PSA, Gleason grade, and clinical stage—parameters that capture tumor burden and differentiation, but not the underlying molecular drivers. This gap has motivated efforts to refine risk stratification using genomic and imaging biomarkers (discussed below). Contemporary staging increasingly incorporates PSMA-targeted positron emission tomography (PET), which detects nodal and distant metastases more accurately than conventional computed tomography and bone scintigraphy and reclassifies a meaningful proportion of patients at diagnosis [13].

### 3.2. Biological and Genomic Heterogeneity

The clinical heterogeneity of high-risk prostate cancer reflects an equally diverse biology. Genomic profiling of localized and metastatic tumors has revealed recurrent alterations in the AR axis, the PI3K-AKT-PTEN pathway, the RB1 and TP53 tumor suppressors, and DNA damage repair genes, as well as ETS-family fusions such as TMPRSS2-ERG [4,8]. The ERG fusion is present in roughly 46% of primary tumors, whereas AR (about 63%) and TP53 (about 53%) alterations dominate metastatic castration-resistant disease [14,15]. The alterations present, and their combinations, differ from patient to patient and shift over the disease course, shaping both the tempo of progression and the response to specific therapies.

Two features matter the most in delaying castration resistance. First, homologous recombination repair (HRR) deficiency—most often BRCA2, but also BRCA1, ATM, and related genes—is enriched in metastatic and high-risk disease and marks a subgroup of clear prognostic and therapeutic significance [8]. Second, combined RB1 and TP53 loss, often with PTEN loss, predisposes to lineage plasticity and treatment-emergent neuroendocrine differentiation, a largely AR-independent route of resistance that remains difficult to treat [4,8].

## 4. The Evolutionary Biology of Castration Resistance

**Figure 1:**
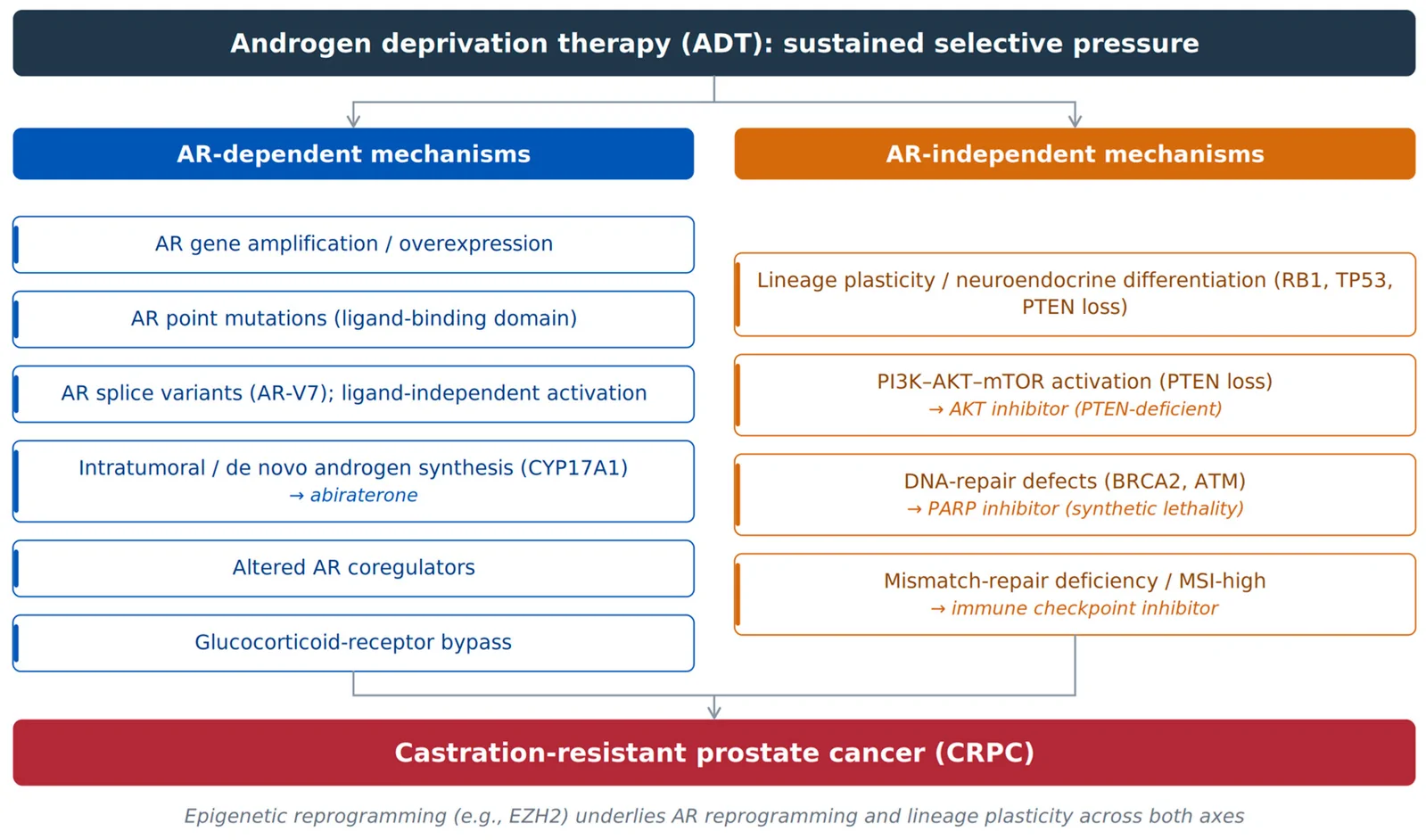
Principal mechanisms of castration resistance in prostate cancer. Under sustained androgen deprivation, tumors progress to castration-resistant disease through AR-dependent mechanisms (**left**) and AR-independent mechanisms (**right**); representative actionable targets are indicated. AR, androgen receptor; AR-V7, AR splice variant 7; MSI, microsatellite instability; PARP, poly(ADP-ribose) polymerase.

Castration resistance arises through a diverse and overlapping set of adaptive mechanisms that allow tumor cells to sustain proliferative and survival signaling despite castrate testosterone levels. These are conventionally divided into mechanisms that reactivate or sustain AR signaling (AR-dependent) and those that bypass the receptor altogether (AR-independent), although the two frequently coexist and interact (Figure 1) [4,8,16]. Therefore, each pathway is double-edged: a route to treatment failure and, at the same time, a potential therapeutic target.

### 4.1. Androgen Receptor-Dependent Mechanisms

Most castration-resistant tumors remain dependent on AR signaling, which they sustain through several overlapping adaptations. In a necropsy series spanning two decades, 85% of castration-resistant tumors were AR-driven in the era before abiraterone and enzalutamide, with 10% neuroendocrine and 5% neither. That majority has since narrowed: in the 2012–2016 era the AR-null, non-neuroendocrine share rose to roughly one fifth [17]. Amplification and overexpression of the AR gene increase receptor abundance and sensitize cells to the low residual androgen that persists during ADT [16]. Point mutations in the AR ligand-binding domain can broaden ligand specificity or convert antagonists and even glucocorticoids into agonists, thereby undermining antiandrogen therapy [4]. Constitutively active splice variants, of which AR-V7 is the best characterized, lack the ligand-binding domain and remain transcriptionally active without ligand; their expression is associated with resistance to abiraterone and enzalutamide [18].

Beyond the receptor itself, tumors can restore intratumoral androgen levels through de novo and adrenal-precursor steroidogenesis, frequently by upregulating CYP17A1—the rationale for the CYP17 inhibitor abiraterone [8,16]. Shifts in the balance between AR coactivators and corepressors further modulate receptor output, and upregulation of the glucocorticoid receptor can substitute for AR by driving an overlapping transcriptional program, contributing to antiandrogen resistance [4,16].

### 4.2. Androgen Receptor-Independent Mechanisms and Lineage Plasticity

A clinically important route of escape abandons AR dependence altogether. Under sustained, potent AR inhibition, a subset of tumors undergoes lineage plasticity—a wholesale shift in cellular identity—away from an AR-driven luminal state toward AR-low or AR-null phenotypes, culminating in treatment-emergent neuroendocrine prostate cancer (NEPC) [4,16]. In a prospective multi-institutional study, NEPC was identified in roughly 17% of metastatic castration-resistant biopsies obtained after AR-pathway inhibition [19]. This is permitted by a characteristic genetic background of combined RB1 and TP53 inactivation, frequently with PTEN loss and MYC activation, which together license cells to exit their lineage [8,20]. The molecular machinery has recently been clarified. The proneural transcription factor ASCL1 acts as a master regulator that, following AR-pathway inhibition, drives large-scale chromatin remodeling and activates stem-like and neuronal transcriptional programs, with additional contributions from FOXA2, NEUROD1, POU2F3, and the pluripotency factors SOX2 and NANOG [20,21]. Lineage-tracing studies show that emergent neuroendocrine cells arise from existing luminal epithelial cells—a genuine change in cell state rather than an outgrowth of a pre-existing neuroendocrine population [20].

These tumors are typically aggressive, respond poorly to AR-directed therapy, and frequently express low levels of PSMA, complicating both treatment and molecular imaging [16]. Lineage-specific targeted approaches remain investigational; the Aurora kinase A inhibitor alisertib, for example, did not meet its primary endpoint in a phase II neuroendocrine prostate cancer trial [22]. Lineage plasticity, however, is dynamic and—at least in preclinical models—reversible. Disrupting ASCL1, a master regulator of the neuroendocrine program, can revert neuroendocrine cells toward a luminal, AR-dependent, castration-sensitive state, and intervention before the transition prevents the switch altogether [20,21]. Targeting the polycomb machinery (EZH2/PRC2) can likewise re-impose differentiation, although current evidence indicates that EZH2 inhibition drives differentiation forward rather than a simple reversal to the luminal lineage [23]. This plasticity makes the neuroendocrine transition a therapeutic target in its own right and points to its regulators as a means of attack (Section 7).

Neuroendocrine transformation is not the only treatment-induced lineage shift. Squamous carcinoma of the prostate is rare, with a reported incidence of at least 1%, and roughly half of reported cases are thought to follow treatment rather than arise de novo [24]. The molecular evidence is confined to individual cases. In the first genomic profiling of a treatment-emergent squamous metastasis, the lesion carried biallelic RB1 loss and showed low PSMA uptake, a combination that would blunt both AR-directed therapy and PSMA-directed imaging [24]. In a separate case, the same TP53 mutation was present in the pre-treatment adenocarcinoma and in the squamous tumor that followed, indicating transformation of the original clone rather than a second primary [25].

Tumors may also sustain growth through survival signaling that runs parallel to, or is in feedback with, the AR axis. The PI3K-AKT-mTOR pathway, commonly activated by PTEN loss, is a prominent example; it engages in reciprocal feedback with AR signaling, such that inhibiting one pathway can relieve the suppression of the other [16]. This crosstalk is the rationale for combined AR and AKT-pathway blockade in PTEN-deficient tumors, now validated in a molecularly selected phase III population (Section 5.1).

### 4.3. DNA Damage Repair Defects and Genomic Instability

Defects in DNA damage repair, particularly HRR deficiency, drive genomic instability, thus creating an actionable vulnerability. About one fifth of metastatic castration-resistant tumors carry a BRCA2, BRCA1 or ATM alteration, and roughly 12% of men with metastatic prostate cancer carry a deleterious germline DNA-repair mutation [15,26]. Tumors with pathogenic BRCA2, BRCA1, ATM, or related alterations are sensitive to poly(ADP-ribose) polymerase (PARP) inhibition through synthetic lethality, which is the basis for olaparib and rucaparib in molecularly selected patients [27]. Beyond blocking single-strand-break repair, PARP inhibitors trap PARP1 on damaged DNA, and the resulting protein–DNA complexes stall advancing replication forks. Collision with a stalled fork generates a double-strand break in any dividing cell. Cells with intact HRR repair the break accurately; cells with HRR deficiency cannot, so the damage is lethal, and this is what makes the synthetic lethality selective for the tumor. Trapping potency differs among agents and may contribute to their differing activity and toxicity [28]. A smaller subset exhibits mismatch-repair deficiency and microsatellite instability, present in 32 of 1033 men (3.1%) with evaluable tumor sequencing in one institutional series [29]. Because defective mismatch repair leaves replication-slippage errors uncorrected, these tumors accumulate large numbers of mutations, including frameshift insertions and deletions that generate entirely novel peptide sequences. Such neopeptides are presented on major histocompatibility complex molecules and can be recognized as foreign by T cells, which is the rationale for immune checkpoint inhibition in this subset [30]. A high mutational burden is not sufficient on its own, since loss of antigen presentation can render the neoantigens invisible [30]. Responses occur but are inconsistent: of the 11 men in that series with mismatch-repair-deficient castration-resistant disease who received checkpoint blockade, 6 had a PSA decline of more than 50%, and 4 of those 6 had radiographic responses [29]. Germline and somatic testing for these alterations is now a standard part of the workup for advanced disease [10].

### 4.4. Epigenetic Reprogramming

Superimposed on genetic alterations, epigenetic mechanisms, such as DNA methylation, histone modifications, and chromatin remodeling, play a central role in shaping the resistant phenotype. Dysregulation of chromatin regulators such as EZH2 contributes to AR reprogramming and to the lineage plasticity underlying neuroendocrine differentiation [16,21]. EZH2 acts in two ways. Canonically, as the catalytic subunit of Polycomb repressive complex 2 (PRC2), it deposits the repressive H3K27me3 mark. In castration-resistant adenocarcinoma, neuroendocrine lineage transcription factors such as ASCL1 carry high levels of that mark and are transcriptionally repressed, so the canonical function holds the neuronal program shut rather than opening it [23]. Non-canonically, and independently of PRC2, phosphorylated EZH2 can act as a transcriptional coactivator of the AR, sustaining AR output rather than silencing it [31]. In neuroendocrine tumors, those same lineage genes carry the activating H3K4me3 mark alongside H3K27me3, so they are poised rather than shut, and inhibiting EZH2 induces them further instead of restoring the luminal lineage [23]. Because epigenetic states are reversible in principle, they are appealing drug targets, and EZH2 inhibitors are being tested with AR-directed therapy, with mixed results so far. In the randomized phase II CELLO-1 study, adding the EZH2 inhibitor tazemetostat to enzalutamide did not improve radiographic progression-free survival (median 16.6 versus 13.8 months; *p* = 0.3704) [32]. In contrast, a randomized dose-expansion cohort of a phase I study found that mevrometostat plus enzalutamide prolonged radiographic progression-free survival over enzalutamide alone (median 14.3 versus 6.2 months; hazard ratio 0.51, 90% CI 0.28–0.95) [33]. That signal is now being tested in the phase III MEVPRO-1 and MEVPRO-2 trials [34].

## 5. Multidisciplinary and Multimodal Strategies to Delay Castration Resistance

**Figure 2:**
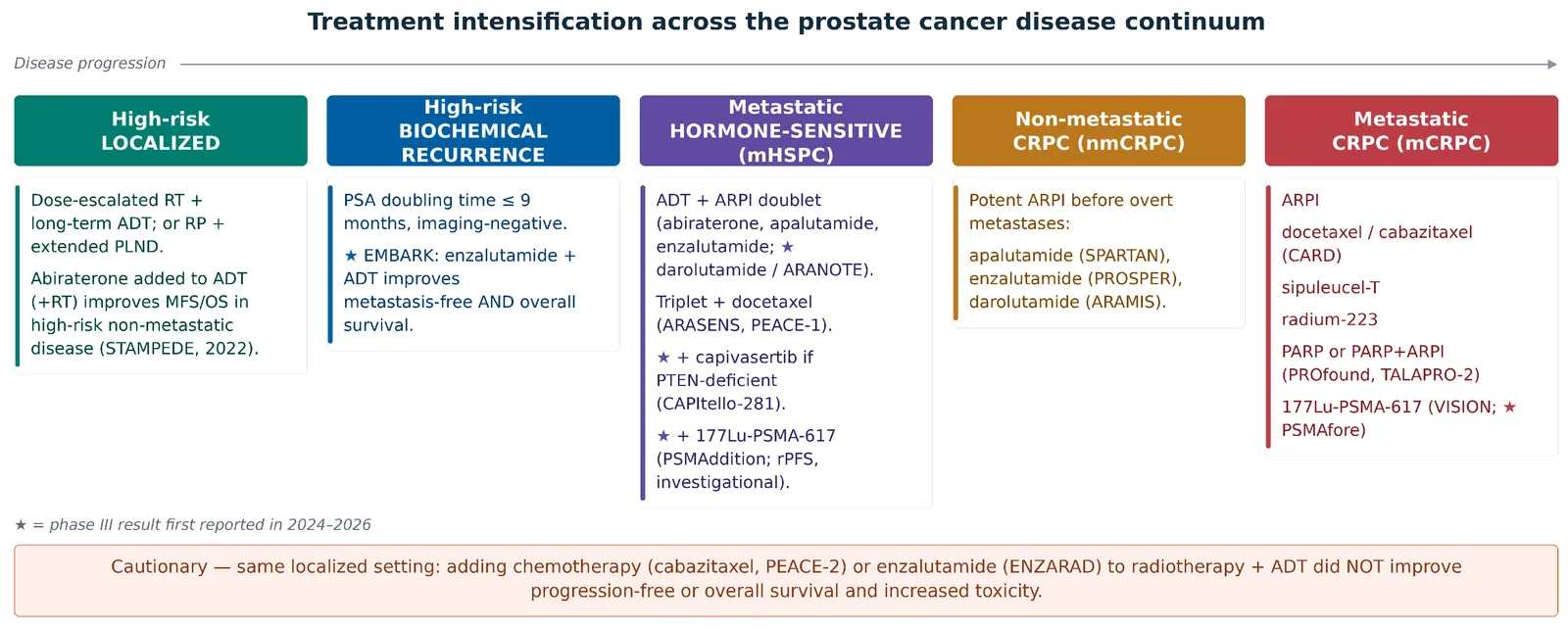
Treatment intensification across the prostate cancer disease continuum. For each disease state, standard and intensified options are shown. A star marks phase III results first reported in 2024–2026; of these, CAPItello-281 and PSMAddition have immature overall survival data and are not yet standard of care (Section 5.1). The STAMPEDE result shown for high-risk non-metastatic disease is reference [35]. ADT, androgen deprivation therapy; ARPI, AR-pathway inhibitor; CRPC, castration-resistant prostate cancer; MFS, metastasis-free survival; mHSPC, metastatic hormone-sensitive prostate cancer; nmCRPC, non-metastatic CRPC; OS, overall survival; PLND, pelvic lymph-node dissection; PSA, prostate-specific antigen; rPFS, radiographic progression-free survival; RP, radical prostatectomy; RT, radiotherapy.

If castration resistance is a product of clonal evolution under therapeutic pressure, delaying it requires strategies that maximize early tumor-cell kill, use mechanisms that are not subject to shared resistance, and limit the selection of resistant clones. In practice this means the coordinated use of systemic therapy, radiation, surgery, and supportive care, applied differently at each disease stage (Figure 2). One caveat applies throughout. These interventions may delay the clinical emergence of castration resistance by reducing tumor burden and limiting clonal selection, but most pivotal trials were not designed with time to CRPC as the primary endpoint. The evidence below is therefore largely interpreted through endpoints such as overall, metastasis-free, and radiographic progression-free survival. We consider each modality in turn.

### 5.1. Systemic Therapy and the Role of Medical Oncology

Over the past decade, intensifying systemic therapy—adding active agents upfront rather than reserving them for sequential use at progression—has consistently improved survival, and the point at which it helps has moved steadily earlier. The most recent extension applies to high-risk biochemical recurrence after primary therapy. In the phase III EMBARK trial, men with a PSA doubling time of nine months or less and no metastases on conventional imaging derived a metastasis-free survival benefit from enzalutamide combined with leuprolide (hazard ratio 0.42) [36]. The final analysis showed a significant overall survival benefit for the combination over leuprolide alone—approximately a 40% reduction in the risk of death, with 8-year overall survival of 78.9% versus 69.5%. By contrast, enzalutamide monotherapy did not significantly improve survival [37]. EMBARK thus establishes intensification as a survival-prolonging strategy before metastases are detectable on conventional imaging.

In metastatic hormone-sensitive prostate cancer (mHSPC), ADT alone is no longer adequate treatment. Adding an AR-pathway inhibitor (LATITUDE [38]; TITAN [39]; ARCHES [40]; ENZAMET [41]; ARANOTE [42]) or docetaxel (CHAARTED [5]; STAMPEDE [6]) to ADT improved radiographic progression-free or overall survival. Triplet therapy with ADT, docetaxel, and an AR-pathway inhibitor improved survival further, particularly in high-volume disease (ARASENS [43]; PEACE-1 [44]). The practical question is therefore not whether to intensify but how far. Each trial was compared with ADT alone rather than with another intensified regimen, so the evidence does not rank these agents against one another. What it does inform is whether to add docetaxel as a third agent, a decision that turns on disease volume and fitness for chemotherapy. Intensifying while tumor burden and clonal diversity are lowest is the principle these trials share.

Two recent trials point beyond uniform intensification, and both are emerging rather than established evidence. Molecular selection now has phase III support. In CAPItello-281, adding the AKT inhibitor capivasertib to abiraterone and ADT in PTEN-deficient de novo mHSPC—a poor-prognosis subgroup comprising roughly one quarter of cases—prolonged radiographic progression-free survival (median 33.2 versus 25.7 months; hazard ratio 0.81). However, overall survival data remain immature [45]. This realizes the AR-AKT co-targeting rationale of Section 4.2, and it succeeds where the unselected approach had not. In IPATential150, ipatasertib added to abiraterone improved radiographic progression-free survival in the PTEN-loss subgroup of metastatic castration-resistant prostate cancer (mCRPC) but not in the all-comers population [46]. Radioligand therapy has moved earlier on similar logic. In PSMAddition, the first phase III trial of radioligand therapy in mHSPC, adding ^177^Lu-PSMA-617 to ADT plus an AR-pathway inhibitor in PSMA-positive disease, improved radiographic progression-free survival (hazard ratio 0.72) consistently across subgroups. No overall survival advantage has yet been shown, quality of life was modestly lower, and higher-grade second primary cancers and renal events were approximately doubled. Radioligand therapy in this setting therefore remains investigational pending longer follow-up and regulatory review [47]. For the clinician the consequence is that PTEN status and PSMA phenotype are becoming selection variables at diagnosis rather than at progression.

Early AR-pathway intensification also benefits patients with high-risk non-metastatic disease. In the STAMPEDE platform, adding abiraterone to ADT (with radiotherapy planned for most patients) improved metastasis-free survival (hazard ratio 0.53) and overall survival, whereas the addition of enzalutamide to abiraterone increased toxicity without further benefit [35]. As discussed in Section 6.2, the success of AR-directed intensification in localized disease contrasts sharply with the failure of chemotherapy-based intensification in the same setting.

In non-metastatic CRPC, apalutamide (SPARTAN [48]), enzalutamide (PROSPER [49]), and darolutamide (ARAMIS [50]) prolong metastasis-free and overall survival, demonstrating the value of potent AR inhibition before overt metastases appear.

By the time disease is castration-resistant, the therapeutic goal is no longer to delay resistance, but to maintain control using mechanistically distinct, non-cross-resistant agents. Several such options carry phase III support (Table 2; trial-level detail in Table S1): taxane chemotherapy (docetaxel and cabazitaxel), AR-pathway inhibitors, the autologous cellular immunotherapy sipuleucel-T [51], the bone-seeking radiopharmaceutical radium-223 [52], PARP inhibitors in HRR-deficient tumors [27], and PSMA radioligand therapy [53,54]. The reach of several has expanded: ^177^Lu-PSMA-617, established after taxane therapy in VISION [53], has also improved radiographic progression-free survival when used before taxane chemotherapy in PSMAfore [55], and PARP inhibition has moved into first-line mCRPC in combination with AR-pathway inhibitors (TALAPRO-2 [56]). Which agent is chosen next matters less than whether its mechanism differs from that of the one that has just failed. Sequential use of AR-pathway inhibitors is associated with limited benefit, likely reflecting cross-resistance. Switching to a non-AR-directed mechanism is therefore generally preferred after progression on a prior agent, as shown in CARD, where cabazitaxel was superior to a second AR-pathway inhibitor after docetaxel and one AR-pathway inhibitor [57].

### 5.2. The Role of Radiation Therapy

Radiation is a curative-intent option for high-risk localized disease and an increasingly important modality across the disease spectrum. For high-risk localized cancer, dose-escalated external-beam radiation with intensity-modulated and image-guided techniques, combined with long-term ADT, is a guideline-endorsed standard; a brachytherapy boost can further improve biochemical control at the cost of increased genitourinary toxicity [10]. The optimal ADT duration is long-term (generally 18–36 months), reflecting the evidence that adequate androgen suppression is integral to the efficacy of radiation [10]. How often radiation is used varies as much with the health system as with the tumor. In a Netherlands Cancer Registry study of 103,059 men with non-metastatic disease, the proportion of men with high-risk localized cancer who received external-beam radiotherapy rose from 37% in 2008 to 45% in 2019 [61]. Among those with locally advanced disease the proportion rose from 50% to 57% over the same period [61]. In intermediate-risk and high-risk disease, the choice between radiotherapy and prostatectomy tracked age, comorbidity, travel time, and the modalities available at the diagnosing hospital [61]. Elsewhere the balance sits differently. In a 2% random sample of the Korean National Health Insurance database, radiotherapy was the primary treatment for 177 of 1382 men actively treated between 2003 and 2013 (12.8%), against 38.8% for surgery and 48.4% for ADT [62]. Its share fluctuated rather than rose, and the odds of radiotherapy relative to surgery fell as the years advanced (adjusted odds ratio 0.93 per year, 95% CI 0.87–0.99) [62]. The investigators suggested that urologist-led diagnosis and private insurance that reimburses inpatient but not outpatient care may both contribute [62]. The two series are not directly comparable, because the Dutch figures are risk-stratified and the Korean figures are not. Together they indicate that referral pathway and local capacity, not tumor biology alone, determine whether radiation is offered.

Radiation has also extended into the metastatic setting. In low-volume mHSPC, radiation to the primary tumor has been shown to improve overall survival, as demonstrated in the prostate-radiotherapy comparison within STAMPEDE [58] and confirmed by a meta-analysis [63]. Surgical cytoreduction of the primary is under investigation: a multicenter phase I trial of cytoreductive radical prostatectomy in node-positive or metastatic disease reported acceptable morbidity and durable responses in a subset of patients, with correlative genomics suggesting molecular determinants of benefit [64]. In oligometastatic disease, metastasis-directed stereotactic ablative radiation delays progression and the need for systemic therapy in randomized trials such as STOMP [65] and ORIOLE [66], reducing the tumor burden while deferring the toxicity of lifelong ADT.

### 5.3. The Contribution of Surgery

Radical prostatectomy with extended pelvic lymph-node dissection plays an established role in selected patients with high-risk localized and locally advanced disease, typically as a component of a multimodal strategy rather than as monotherapy [10]. It provides durable local control, complete pathologic staging, and prognostic information that can guide the use or de-intensification of adjuvant radiation and systemic therapy. A substantial proportion of high-risk patients are found to have specimen-confined disease at pathology, and post-prostatectomy pathology together with PSA kinetics defines risk groups that inform the need for, and timing of, salvage therapy [67]. Guidelines, including those of the EAU, support radical prostatectomy in this setting as part of a multimodal approach, while acknowledging that randomized data directly comparing surgery with radiation plus ADT are still maturing, a question addressed by trials such as SPCG-15 [68]. Neoadjuvant chemohormonal therapy (docetaxel plus ADT, in CALGB 90203) did not meet its primary endpoint of 3-year biochemical progression-free survival; however, it did improve pathologic features at prostatectomy and was associated with suggestive longer-term benefits, so its role remains investigational [69].

### 5.4. Integrating Supportive and Survivorship Care

Because high-risk prostate cancer is frequently managed with prolonged androgen suppression, supportive care is integral rather than ancillary. ADT is associated with accelerated bone loss and fracture risk, adverse metabolic and cardiovascular effects, sexual dysfunction, vasomotor symptoms, fatigue, sarcopenia, and cognitive effects, all of which affect quality of life and treatment adherence [10]. Proactive management—monitoring and preserving bone mineral density, using bone-targeted agents such as denosumab or zoledronic acid to reduce skeletal-related events in patients with bone metastases, assessing cardiovascular risk, prescribing structured exercise, and managing metabolic effects—mitigates these harms [10]. Embedding supportive care, symptom management, and shared decision-making in the treatment pathway helps sustain patients through the prolonged and intensified regimens that are increasingly standard [10].

### 5.5. Sequencing, Integration, and the Multidisciplinary Team

These modalities are most effective when integrated rather than applied in isolation. Decisions regarding local therapy (surgery versus radiation), the combination and sequence of systemic agents, the timing of metastasis-directed treatment, and the scheduling of molecular testing and imaging are inherently multidisciplinary. A coordinated team—urologists, radiation oncologists, medical oncologists, pathologists, radiologists and nuclear-medicine physicians, and supportive-care specialists—is best positioned to match the intensity and sequence of therapy to tumor biology, disease stage, and patient circumstances. Coordinating non-cross-resistant therapies in a rational sequence is how the team maximizes disease control while limiting the selection of resistant clones.

Agreeing on this in principle is not the same as achieving it in routine practice. A stepped-wedge cluster-randomized implementation trial across nine Australian hospitals separated the two. A pathology-led process that flagged adverse prostatectomy specimens for the team meeting raised the proportion of patients discussed from 17% to 59% (adjusted relative risk 4.32). Referral to radiation oncology nonetheless did not change (32% versus 30%), and only 62 of 140 patients whom the team recommended for referral were referred within four months [70]. Structured case-finding changes what the team sees; it does not by itself change what the treating clinician does. Two measures follow from this. The first is to define in advance which patients are routed to the team, rather than leaving presentation to individual discretion. The second is to record the recommendation in the medical record, so that it is available at the point of care and auditable afterwards [70]. Sequencing decisions depend most on this discipline, because they are made repeatedly over years and each one is constrained by what was given before; the non-cross-resistance principle is operative only if the team can see the whole treatment history. The documented underuse of guideline-recommended intensification in routine mHSPC care [71] is what these steps are meant to prevent.

## 6. Lessons from Pivotal Trials: What Works and What Does Not

The most reliable guidance for delaying castration resistance comes from the pattern of results across pivotal trials, rather than from individual case reports. Not all trials carry equal weight; it helps to read them according to their level of evidence. Practice-changing evidence includes EMBARK in high-risk biochemical recurrence [36,37], doublet and triplet intensification in mHSPC (e.g., ARASENS [43] and PEACE-1 [44]), AR-pathway intensification in high-risk non-metastatic disease (STAMPEDE [35]), and ^177^Lu-PSMA-617 after taxane therapy (VISION [53]). Cautionary or negative evidence includes the failure of chemotherapy intensification (PEACE-2 [59]) and enzalutamide added to radiotherapy (ENZARAD [60]) in localized disease and the limited activity of immune checkpoint inhibitor monotherapy in unselected mCRPC [16]. Investigational or immature evidence includes radioligand therapy in the hormone-sensitive setting (PSMAddition [47]), AR-AKT co-targeting with immature survival data (CAPItello-281 [45]), and agents directed at lineage plasticity. Two broad lessons follow: some intensification strategies reproducibly improve outcomes; others provide limited or no benefit.

### 6.1. Successful Multimodal Strategies

The most reproducible benefit is seen with early, combined use of mechanistically distinct agents. That benefit runs from high-risk biochemical recurrence (EMBARK [36,37]) through metastatic hormone-sensitive disease, where doublet and triplet regimens consistently outperformed ADT alone [5,6,38,39,40,41,42,43,44] and the biomarker-directed addition of an AKT inhibitor benefited PTEN-deficient tumors [45]. In high-risk disease, combining local therapy (radiation or surgery) with durable and, where indicated, intensified systemic therapy outperformed either modality alone [7,10,35], and in oligometastatic disease, metastasis-directed therapy delayed progression and deferred systemic treatment [65,66]. The common rationale is to reduce tumor burden and clonal diversity before resistance emerges, an interpretation supported by these outcomes, although time to CRPC was generally not the primary endpoint.

### 6.2. Cautionary and Negative Signals

Other strategies have underperformed. Immune checkpoint inhibitor monotherapy, despite its impact in other malignancies, has shown little benefit in unselected metastatic CRPC, reflecting the immunologically cold microenvironment of most prostate cancers; meaningful responses are largely confined to small subgroups with mismatch-repair deficiency, microsatellite instability, or high tumor mutational burden [16]. Sequential AR-pathway inhibition is similarly disappointing, consistent with cross-resistance [57].

A more nuanced lesson concerns which intensification strategies help in localized disease, where the benefit depends on both the agent and the patient. AR-pathway intensification with abiraterone improves metastasis-free and overall survival when added to ADT and radiotherapy in high-risk non-metastatic disease (Section 5.1) [35]. In contrast, cytotoxic chemotherapy has repeatedly failed in this setting. Adding cabazitaxel to radiotherapy and ADT in node-negative, very-high-risk localized disease did not improve clinical progression-free, metastasis-free, or overall survival in PEACE-2 (clinical progression-free survival hazard ratio of approximately 1.1) and increased grade 3 or higher toxicity [59]. This mirrors the earlier failure of docetaxel intensification in non-metastatic STAMPEDE. Enzalutamide added to ADT and radiotherapy likewise did not improve metastasis-free survival in ENZARAD (hazard ratio 0.88) [60]. The lesson is not that localized disease should never be intensified, but that the choice of agent and the patient’s risk profile are decisive. Prostate-cancer-specific survival in PEACE-2 exceeded 89% at nine years across all arms. This led the investigators to question whether node-negative disease currently labeled as very-high-risk is as lethal as the designation implies, a concern amplified by the more accurate staging now afforded by PSMA-PET, which reclassifies a meaningful proportion of such patients [59].

Both overtreatment and undertreatment represent failures of optimal care that the multidisciplinary model is intended to prevent: overtreatment of patients destined for favorable outcomes, as the localized-disease chemotherapy trials suggest, and undertreatment, as exemplified by the documented underuse of guideline-recommended intensification in routine mHSPC practice [71]. Therefore, delaying castration resistance depends on biology- and stage-informed selection and sequencing of effective therapies, rather than on indiscriminate intensification.

## 7. Future Directions

### 7.1. Emerging Therapeutic Strategies

Several strategies may further delay or overcome castration resistance. Some that were recently investigational now have phase III data: PSMA radioligand therapy has moved into earlier disease (PSMAfore [55]; PSMAddition [47]) and AR-AKT co-targeting has been tested in a molecularly selected population (CAPItello-281 [45]). In the hormone-sensitive setting, the survival data from both remain immature, and neither regimen is yet standard of care [45,47]. Next-generation AR-targeting agents are also advancing: AR degraders such as proteolysis-targeting chimeras (PROTACs) and inhibitors of the AR amino-terminal domain aim to suppress AR signaling, even in tumors with AR amplification, ligand-binding-domain mutations, or splice variants that escape current AR-pathway inhibitors [16]. PARP inhibitors continue to expand, both as molecularly selected monotherapy and in combination with AR-pathway inhibitors in earlier disease [27,56]. Immune-based approaches tailored to the cold prostate microenvironment, including bispecific T-cell engagers and chimeric antigen receptor T cells directed against PSMA or STEAP1, are in active clinical development [16]. Neuroendocrine disease is where this progress runs out. No phase III trial has been reported in neuroendocrine prostate cancer, and the evidence base is early-phase, including the negative alisertib trial described in Section 4.2. Tarlatamab, a bispecific T-cell engager directed against delta-like ligand 3 (DLL3), produced objective responses in 10.5% of 38 evaluable patients in the phase Ib DeLLpro-300 study [72]. Every response occurred in the 18 patients with DLL3-positive tumors, among whom the response rate was 22.2%; no patient with a DLL3-negative or unassessed tumor responded [72]. The route of escape that is hardest to treat is therefore also the one supported by the weakest evidence.

One line of work speaks directly to this review’s theme: targeting lineage plasticity itself. The transition to an AR-independent, neuroendocrine state is driven by definable regulators and appears at least partly reversible in preclinical models; therefore, agents disrupting these programs may prevent, delay, or reverse one of the most treatment-refractory routes of escape. The reversal evidence is strongest for ASCL1 [20,21]. EZH2 inhibition is the approach furthest along in the clinic, but early-phase results are mixed. Tazemetostat added to enzalutamide did not improve radiographic progression-free survival [32], whereas mevrometostat did in a randomized dose-expansion cohort [33]. The phase III MEVPRO-1 and MEVPRO-2 trials are under way [34]. Clinical translation would mean reversing an established resistance mechanism rather than merely delaying it, and no trial has yet demonstrated such a reversal.

### 7.2. Biomarker-Driven Personalization

Management is moving toward matching therapy to each tumor’s molecular and phenotypic profile. PSMA-PET has already improved the accuracy of staging and patient selection and is reshaping the definitions of disease extent at diagnosis and recurrence [13]. Liquid biopsy—including circulating tumor DNA and detection of AR alterations such as AR-V7—offers a minimally invasive means of tracking clonal evolution and anticipating resistance [18]. Germline and somatic testing for DNA damage repair defects, immunohistochemical and genomic assessment of PTEN, and tissue-based genomic classifiers increasingly identify patients who will benefit from PARP inhibition, AKT-pathway blockade, immune checkpoint inhibition, or intensified therapy, and spare others from unnecessary treatment [10]. Even conventional biomarkers retain prognostic value. After definitive radiotherapy, the depth of the PSA nadir and the time to reach it stratify the risk of biochemical and clinical failure in intermediate- and high-risk localized disease [73]. In bone-metastatic disease, the PSA response to androgen deprivation together with serum alkaline phosphatase predicts survival more reliably than baseline PSA alone [74]. When integrated into routine practice, these tools should enable treatment that anticipates rather than reacts to the evolution of the disease.

### 7.3. Open Questions

Important uncertainties remain. The optimal sequencing and combination of the expanding array of active agents are incompletely defined, as is how best to balance maximal upfront intensification against cumulative toxicity, cost, and quality of life. The failure of chemotherapy intensification in very-high-risk localized disease is set against the benefit of AR-pathway intensification in the same setting and the high cancer-specific survival observed regardless of treatment. This contrast underscores the need for better tools to distinguish patients who require intensified therapy from those who do not, a need that molecular and imaging biomarkers are beginning to address. Whether lineage-plasticity strategies can be translated from preclinical models into clinical benefit is among the most consequential open questions in this field. Addressing these issues will require well-designed, biomarker-stratified trials and continued multidisciplinary collaboration.

## 8. Discussion

The trials of recent years support a consistent but qualified conclusion. Intensification improves outcomes, and increasingly early in the disease course, but unevenly and not with every agent. The high cancer-specific survival of some node-negative, very-high-risk localized cohorts even raises the possibility that a subset of these patients is intensified for a risk that would never have become clinically meaningful. Therefore, the central question is less whether to intensify than whom to intensify, and with what—a problem of patient selection, in which biology, imaging, and clinical judgment must converge.

Trial populations also differ systematically from real-world patients. Phase III cohorts are generally younger, fitter, and better supported than many men seen in routine practice, where comorbidity, frailty, and limited social support constrain what is feasible. Triplet therapy and prolonged AR-pathway intensification impose a considerable burden—years of androgen suppression combined with an additional agent and its metabolic, cardiovascular, and musculoskeletal effects—and adherence over this horizon is not assured. A regimen discontinued prematurely delivers neither the survival benefit observed in trials nor the quality of life of less intensive treatment. This argues for treating toxicity management, symptom control, and shared decision-making as seriously as the choice of agent. The documented underuse of guideline-recommended intensification in routine mHSPC care [71] suggests that the gap between evidence and practice is already substantial.

Access and cost shape these decisions in ways that vary according to the health system, as the national differences in radiotherapy use already show (Section 5.2). In Korea, PSMA-PET is reimbursed for the staging of higher-risk disease and for restaging at biochemical recurrence, so the molecular imaging underpinning modern selection is, for our patients, routinely available; the staging refinements discussed throughout this review are part of standard practice. PSMA-targeted radioligand therapy is a different matter: despite its phase III support, it is not yet reimbursed in Korea, carries a substantial out-of-pocket cost, and can be delivered only at centers with appropriate nuclear-medicine licensing, radiation-handling facilities, and trained personnel. Therefore, in our setting, patients who may benefit from radioligand therapy can often be identified more readily than they can be treated. The cost of triplet regimens, next-generation AR-pathway inhibitors, and repeated molecular testing similarly bears on real decisions, and any individualized strategy must be matched to the resources actually available, not only to the tumor.

This review has several limitations. It is a narrative synthesis rather than a systematic review or meta-analysis: no formal protocol, registered search strategy, or quality grading was applied, and source selection reflects the authors’ focus on the multidisciplinary management of high-risk disease. We restricted ourselves to English-language sources and weighted randomized phase III data and major guidelines most heavily, which may underrepresent the findings reported elsewhere. The field is evolving rapidly, and several trials on which we rely, particularly in radioligand therapy and AR-AKT co-targeting, have immature overall survival data; conclusions based on progression-free endpoints should be regarded as provisional. Finally, the evolutionary framing adopted here is a conceptual framework, not an evidence-based therapeutic strategy. No trial has tested whether therapy selected to shape clonal evolution outperforms the same therapy selected by stage, risk group, and biomarker. Time to castration resistance was also not the primary endpoint of the trials on which the framing rests (Section 5). It earns its place by organizing the evidence and by making the reasons for early, mechanistically diverse treatment explicit, not by supplying decisions of its own. Read as more than that, it would credit the concept with results that belong to the individual trials.

## 9. Conclusions

High-risk prostate cancer is defined, above all, by its evolutionary potential—its capacity to adapt, under therapeutic pressure, toward castration resistance through a diverse repertoire of AR-dependent and AR-independent mechanisms. Understood as a conceptual framework rather than an evidence-based therapeutic strategy, this evolutionary framing points clinical decisions toward acting early, combining mechanisms not subject to shared resistance, and matching treatment intensity to tumor biology and disease stage. The benefit of intensification is real, but it is not uniform. It is demonstrable from high-risk biochemical recurrence through metastatic hormone-sensitive and castration-resistant disease, and it extends to high-risk non-metastatic disease through AR-pathway intensification; however, it remains stage- and agent-dependent, as shown by the failure of chemotherapy intensification in very-high-risk localized disease. The most reproducible successes—early intensification, combined local and systemic therapy, biomarker-directed treatment, and metastasis-directed therapy in oligometastatic disease—share the rationale for reducing tumor burden and clonal diversity before resistance emerges, while avoiding both overtreatment and undertreatment. Emerging therapies, particularly those targeting the potentially reversible biology of lineage plasticity, raise the prospect of moving beyond delay toward reversal of established resistance, although this possibility remains investigational and has not yet been demonstrated clinically. Realizing these goals depends on integrated, biology-informed multidisciplinary care that adapts the intensity, combination, and sequence of therapy to each patient as the disease evolves.

## Figures and Tables

**Table 1:** Representative criteria for classifying high-risk, very-high-risk, and locally advanced prostate cancer.

Risk Category	Defining Criteria (Any One Feature)	Source/Notes
High-risk localized	PSA > 20 ng/mL; or ISUP grade group 4–5 (Gleason score 8–10); or clinical stage ≥ cT2c (D’Amico) or ≥cT3a (NCCN)	D’Amico; NCCN [9]; EAU [10] (EAU defines by grade group 4–5 or PSA > 20; cT3–cT4/cN+ classified as locally advanced)
Very-high-risk localized	Clinical stage cT3b–cT4; or primary Gleason pattern 5; or >4 biopsy cores of grade group 4–5; or multiple high-risk features	NCCN [9]
Locally advanced	Clinical stage cT3–cT4; or regional nodal involvement (cN1)	EAU [10]

Abbreviations: ISUP, International Society of Urological Pathology; NCCN, National Comprehensive Cancer Network; EAU, European Association of Urology.

**Table 2:** Selected phase III trials defining treatment intensification across the prostate cancer disease continuum.

Setting	Trial	Intervention	Key Outcome (HR; 95% CI)
High-risk biochemical recurrence (PSA doubling time ≤ 9 months)	EMBARK [36,37]	Enzalutamide + leuprolide	MFS HR 0.42 (0.30–0.61); OS improved (8-year 78.9% vs. 69.5%)
mHSPC	LATITUDE [38]; TITAN [39]; ARCHES [40]; ENZAMET [41]; ARANOTE [42]	AR-pathway inhibitor + ADT: abiraterone (LATITUDE), apalutamide (TITAN), enzalutamide (ARCHES, ENZAMET), darolutamide (ARANOTE)	OS HR 0.62 (0.51–0.76), 0.65 (0.53–0.79), and 0.67 (0.52–0.86) in LATITUDE, TITAN, and ENZAMET; rPFS HR 0.39 (ARCHES) and 0.54 (0.41–0.71) (ARANOTE)
mHSPC	CHAARTED [5]; STAMPEDE [6]	Docetaxel + ADT	OS HR 0.61 (0.47–0.80), greatest in high-volume disease; OS HR 0.78 (0.66–0.93)
mHSPC	ARASENS [43]; PEACE-1 [44]	Triplet: ADT + docetaxel + darolutamide (ARASENS) or abiraterone (PEACE-1)	OS HR 0.68 (0.57–0.80); PEACE-1 docetaxel (triplet) population OS HR 0.75, rPFS HR 0.50, overall population OS HR 0.82 (0.69–0.98)
mHSPC, PTEN-deficient	CAPItello-281 [45]	Capivasertib + abiraterone + ADT	rPFS HR 0.81 (0.66–0.98); OS immature
mHSPC, PSMA-positive	PSMAddition [47]	^177^Lu-PSMA-617 + ADT + ARPI	rPFS HR 0.72 (0.58–0.90); OS immature; investigational
Low-volume mHSPC	STAMPEDE arm H [58]	Prostate radiotherapy	OS HR 0.68 (0.52–0.90)
High-risk non-metastatic (M0)	STAMPEDE [35]	Abiraterone + ADT (±radiotherapy)	MFS HR 0.53 (0.44–0.64); OS HR 0.60
nmCRPC	SPARTAN [48]; PROSPER [49]; ARAMIS [50]	Apalutamide (SPARTAN), enzalutamide (PROSPER), or darolutamide (ARAMIS)	MFS HR 0.28 (0.23–0.35), 0.29 (0.24–0.35), and 0.41 (0.34–0.50)
mCRPC, PSMA-positive	VISION [53]; PSMAfore [55]	^177^Lu-PSMA-617 after taxane (VISION) or before taxane (PSMAfore)	OS HR 0.62 (0.52–0.74); rPFS HR 0.41 (0.29–0.56)
mCRPC, HRR-mutated and first-line	PROfound [27]; TALAPRO-2 [56]	Olaparib (PROfound); talazoparib + enzalutamide (TALAPRO-2)	rPFS HR 0.34 (0.25–0.47) in the BRCA/ATM cohort; rPFS HR 0.63 (0.51–0.78)
mCRPC	IMPACT [51]; ALSYMPCA [52]	Sipuleucel-T (IMPACT); radium-223 (ALSYMPCA)	OS HR 0.78 (0.61–0.98); OS HR 0.70 (0.58–0.83)
Very-high-risk localized	PEACE-2 [59]	Cabazitaxel added to RT + ADT	No benefit: cPFS HR 1.09 (0.85–1.38); more toxicity
High-risk localized	ENZARAD [60]	Enzalutamide added to RT + ADT	No benefit: MFS HR 0.88 (0.67–1.15)

Inclusion is restricted to randomized phase III trials in the disease states covered by this review; trial-level detail, together with the randomized phase II trials (TheraP, STOMP, ORIOLE) and the randomized phase IV CARD trial, is given in Table S1. No phase III trial has been reported in neuroendocrine prostate cancer (Section 7.1). Abbreviations: ADT, androgen deprivation therapy; ARPI, AR-pathway inhibitor; CI, confidence interval; cPFS, clinical progression-free survival; HR, hazard ratio; HRR, homologous recombination repair; MFS, metastasis-free survival; mHSPC, metastatic hormone-sensitive prostate cancer; mCRPC, metastatic castration-resistant prostate cancer; nmCRPC, non-metastatic castration-resistant prostate cancer; OS, overall survival; PSA, prostate-specific antigen; rPFS, radiographic progression-free survival.

## Data Availability

No new data were created or analyzed in this study. Data sharing is not applicable to this article.

## Supplementary Materials

The following supporting information can be downloaded at: https://www.mdpi.com/article/10.3390/jcm15166488/s1. Table S1: Trial-level detail for the randomized trials summarized in Table 2. All trials are randomized phase III except TheraP, STOMP, and ORIOLE, which are randomized phase II, and CARD, which is randomized phase IV.

## Abbreviations

The following abbreviations are used in this manuscript:

ADT	Androgen Deprivation Therapy
AR	Androgen Receptor
AR-V7	Androgen Receptor splice Variant 7
CRPC	Castration-Resistant Prostate Cancer
mCRPC	metastatic Castration-Resistant Prostate Cancer
mHSPC	metastatic Hormone-Sensitive Prostate Cancer
NCCN	National Comprehensive Cancer Network
EAU	European Association of Urology
ISUP	International Society of Urological Pathology
NEPC	Neuroendocrine Prostate Cancer
PARP	Poly(ADP-ribose) Polymerase
PRC2	Polycomb Repressive Complex 2
PROTAC	Proteolysis-Targeting Chimera
PSA	Prostate-Specific Antigen
PSMA	Prostate-Specific Membrane Antigen
PET	Positron Emission Tomography
HRR	homologous recombination repair
DLL3	Delta-Like Ligand 3
