# Supplementary material for: Reprogramming the Evolution of High-Risk Prostate Cancer: Multidisciplinary Strategies to Delay Castration Resistance
Source: J Clin Med. 2026 Aug 21;15(16):6488. doi: 10.3390/jcm15166488 (PMC13514596; doi:10.3390/jcm15166488)
Supplement: Supplementary file 1 [file jcm-15-06488-s001.zip › jcm-4458156-supplementary.pdf]

**Table S1.** Trial-level detail for the randomized trials summarized in Table 2. All trials are randomized phase III except TheraP, STOMP, and ORIOLE, which are randomized phase II, and CARD, which is randomized phase IV.

| Setting | Trial | Intervention | Key outcome (HR; 95% CI) |
|---|---|---|---|
| High-risk biochemical recurrence (PSA doubling time ≤ 9 mo) | EMBARK [36,37] | Enzalutamide + leuprolide | MFS HR 0.42 (0.30–0.61); OS improved (8-yr 78.9% vs 69.5%) |
| mHSPC | CHAARTED [5] | Docetaxel + ADT | OS HR 0.61 (0.47–0.80); greatest in high-volume |
| mHSPC | STAMPEDE [6] | Docetaxel + ADT | OS HR 0.78 (0.66–0.93) |
| mHSPC | LATITUDE [38] | Abiraterone + ADT | OS HR 0.62 (0.51–0.76) |
| mHSPC | TITAN [39] | Apalutamide + ADT | OS HR 0.65 (0.53–0.79) |
| mHSPC | ARCHES / ENZAMET [40,41] | Enzalutamide + ADT | rPFS HR 0.39 (ARCHES); OS HR 0.67 (0.52–0.86) (ENZAMET) |
| mHSPC | ARANOTE [42] | Darolutamide + ADT (doublet) | rPFS HR 0.54 (0.41–0.71) |
| mHSPC | ARASENS [43] | Darolutamide + docetaxel + ADT (triplet) | OS HR 0.68 (0.57–0.80) |
| mHSPC | PEACE-1 [44] | Abiraterone added to ADT + docetaxel (triplet) | Docetaxel (triplet) population: OS HR 0.75, rPFS HR 0.50; overall population OS HR 0.82 (0.69–0.98) |
| mHSPC, PTEN-deficient | CAPItello-281 [45] | Capivasertib + abiraterone + ADT | rPFS HR 0.81 (0.66–0.98); OS immature |
| mHSPC, PSMA-positive | PSMAddition [47] | 177Lu-PSMA-617 + ADT + ARPI | rPFS HR 0.72 (0.58–0.90); OS immature; investigational |
| High-risk non-metastatic (M0) | STAMPEDE [35] | Abiraterone + ADT (± radiotherapy) | MFS HR 0.53 (0.44–0.64); OS HR 0.60 |
| nmCRPC | SPARTAN [48] | Apalutamide | MFS HR 0.28 (0.23–0.35) |
| nmCRPC | PROSPER [49] | Enzalutamide | MFS HR 0.29 (0.24–0.35) |
| nmCRPC | ARAMIS [50] | Darolutamide | MFS HR 0.41 (0.34–0.50) |
| mCRPC | IMPACT [51] | Sipuleucel-T | OS HR 0.78 (0.61–0.98) |
| mCRPC | ALSYMPCA [52] | Radium-223 | OS HR 0.70 (0.58–0.83) |
| mCRPC, HRR-mutated | PROfound [27] | Olaparib | rPFS HR 0.34 (0.25–0.47) (BRCA/ATM cohort) |
| mCRPC, PSMA-positive (post-taxane) | VISION [53] | 177Lu-PSMA-617 | OS HR 0.62 (0.52–0.74) |
| mCRPC | TheraP [54] | 177Lu-PSMA-617 vs cabazitaxel | PSA50 response 66% vs 37%; no OS difference |
| mCRPC, pre-taxane | PSMAfore [55] | 177Lu-PSMA-617 vs ARPI change | rPFS HR 0.41 (0.29–0.56) |
| mCRPC, first-line | TALAPRO-2 [56] | Talazoparib + enzalutamide | rPFS HR 0.63 (0.51–0.78) |
| mCRPC | CARD [57] | Cabazitaxel vs 2nd ARPI | PFS HR 0.54 (0.40–0.73); OS benefit |
| Low-volume mHSPC | STAMPEDE arm H [58] | Prostate radiotherapy | OS HR 0.68 (0.52–0.90) |
| Oligometastatic | STOMP / ORIOLE [65,66] | Metastasis-directed SABR | Delayed progression; ORIOLE 6-mo progression 19% vs 61% (phase II) |

| | | | |
|---|---|---|---|
| Very-high-risk localized | PEACE-2 [59] | Cabazitaxel added to RT + ADT | No benefit: cPFS HR 1.09 (0.85–1.38); more toxicity |
| High-risk localized | ENZARAD [60] | Enzalutamide added to RT + ADT | No benefit: MFS HR 0.88 (0.67–1.15) |

Abbreviations: ADT, androgen deprivation therapy; ARPI, AR-pathway inhibitor; cPFS, clinical progression-free survival; HRR, homologous recombination repair; MFS, metastasis-free survival; mHSPC, metastatic hormone-sensitive prostate cancer; (n)mCRPC, (non-)metastatic castration-resistant prostate cancer; OS, overall survival; PSA, prostate-specific antigen; rPFS, radiographic progression-free survival; SABR, stereotactic ablative radiotherapy.
